# *CaLRR-RLK1,* a novel RD receptor-like kinase from *Capsicum annuum* and transcriptionally activated by CaHDZ27, act as positive regulator in *Ralstonia solanacearum* resistance

**DOI:** 10.1186/s12870-018-1609-6

**Published:** 2019-01-17

**Authors:** Shaoliang Mou, Feng Gao, Lei Shen, Sheng Yang, Weihong He, Wei Cheng, Yang Wu, Shuilin He

**Affiliations:** 1 College of Life Science, Fujian Agriculture and Forestry University, Fuzhou, Fujian 350002 People’s Republic of China; 2 Key Laboratory of Applied Genetics of Universities in Fujian Province, Fujian Agriculture and Forestry University, Fuzhou, Fujian 350002 People’s Republic of China; 3 National Education Ministry Key Laboratory of Plant Genetic Improvement and Comprehensive Utilization, Fujian Agriculture and Forestry University, Fuzhou, Fujian 350002 People’s Republic of China; 4 College of Crop Science, Fujian Agriculture and Forestry University, Fuzhou, Fujian 350002 People’s Republic of China; 5 College of Life Science, Jinggangshan University, Ji’an, Jiangxi 343000 People’s Republic of China

**Keywords:** CaLRR-RLK1, CaHDZ27, Pepper, *Ralstonia solanacearum*, Plant immunity

## Abstract

**Background:**

Bacterial wilt caused by *Ralstonia solanacearum* is one of the most important diseases in pepper worldwide, however, the molecular mechanism underlying pepper resistance to bacterial wilt remains poorly understood.

**Results:**

Herein, a novel RD leucine-rich repeat receptor-like kinase, CaLRR-RLK1, was functionally characterized in immunity against *R. solanacearum*. CaLRR-RLK1 was targeted exclusively to plasma membrane and was up-regulated by *R. solanacearum* inoculation (RSI) as well as by the exogenous application of salicylic acid (SA), methyl jasmonate (MeJA) or ethephon (ETH). The silencing of *CaLRR-RLK1* led to enhanced susceptibility of pepper plants to RSI, accompanied by down-regulation of immunity-related genes including *CaACO*1, *CaHIR1*, *CaPR4* and *CaPO2*. In contrast, transient overexpression of *CaLRR-RLK1* triggered hypersensitive response (HR)-like cell death and H2O2 accumulation in pepper leaves, manifested by darker trypan blue and DAB staining respectively. In addition, the ectopic overexpression of *CaLRR-RLK1* in tobacco plants enhanced resistance *R. solanacearum,* accompanied with the immunity associated marker genes including *NtPR2*, *NtPR2*, *NtHSR203* and *NtHSR515*. Furthermore, it was found that CaHDZ27, a positive regulator in pepper response to RSI in our previous study, transcriptionally activated *CaLRR-RLK1* by direct targeting its promoter probably in a CAATTATTG dependent manner.

**Conclusion:**

The study revealed that *CaLRR-RLK1* confers pepper resistance to *R. solanacearum* as the direct targeting of CaHDZ27.

**Electronic supplementary material:**

The online version of this article (10.1186/s12870-018-1609-6) contains supplementary material, which is available to authorized users.

## Background

Bacterial wilt, caused by *Ralstonia solanacearum* [1], is a highly destructive vascular disease in pepper (*Capsicum annuum*), a solanaceous vegetable of great agricultural importance. It is believed that the most efficient way to cope with such kind of disease is to develop crop variety with high level of disease resistance, a better understanding of molecular basis of pepper resistance to *R. solanacearum* will benefit its genetic improvement.

Upon challenge by pathogens, plant employ two interconnected layers of immunity including pathogen-associated molecular pattern (PAMP)-triggered immunity (PTI) and effector-triggered immunity (ETI) to protect them [2, 3]. Plant perceive exogenous PAMPs derived from microbial pathogens by cell surface-localized proteins, known as pattern recognition receptors (PRRs), which results in PTI. In addition, PRRs can also activate immune response by recognizing the endogenous damage-associated molecular patterns (DAMPs), which are produced as a consequence of pathogen infection and perception [4]. The adapted pathogen develop effectors, which are secreted and injected into host cells where they repress PTI by targeting specific signaling components, leading effectors triggered susceptivity (ETS) [5, 6]. PTI, also called basal defense, is required but not sufficient for disease resistance. Under the selection pressure of ETS, plant might evolve specific receptors, typically resistance (R) proteins, to detect the effectors and result in ETI, which is often accompanied with hypersensitive reaction (HR) and systematic acquired resistance (SAR). Although ETI is more robust, intensive and more directly associated with transcriptional regulation of defense gene expression than PTI, overlapping downstream molecular events have been found between PTI and ETI [7].

Leucine-rich repeat receptor-like protein kinases (LRR-RLKs) are typical plant cell surface-localized PRRs, they contain an extracellular domain with up to 30 leucine-rich repeat (LRRs) to perceive ligands including small molecules, peptides, and entire proteins [8, 9], a TMD (trans-membrane domain) for its cell membrane targeting and an intracellular serine/threonine (Ser/Thr) kinase domain to phosphorylate specific substrates for the signal transduction. Approximately 223 LRR-RLKs in *Arabidopsis* [10, 11], 226 in *Zea mays* [12], 234 in tomato [13], 379 in populous [14], 303 in *Brassica rapa* [15], 188 in pepper [16] have been identified. Some LRR-RLKs have been implicated to regulate plant innate immunity [17–19]. For example, a well-studied PRR LAGELLIN SENSITIVE 2 (FLS2) recognizes and binds a 22-amino acid epitope of bacterial flagellin called flg22 [20, 21]. Upon perception of flg22, FLS2 interacts with another LRR-RLK, BRI1-ASSOCIATED RECEPTOR KINASE 1 (BAK1) [22, 23], and phosphorylates the NADPH oxidase RbohD by association with a receptor-like cytoplasmic kinase (RLCK), BOTRYTIS-INDUCED KINASE 1 (BIK1) [24, 25], leading to a calcium burst and reactive oxygen species (ROS) production, and thereby mounts PAMP-triggered immunity (PTI). By sensing and binding Ax21 derived from pathogen, XA21 confers broad-spectrum resistance to the bacterial disease caused by the pathogen of *Xanthomonas oryzae* pv. *oryzae* (*Xoo*) [26, 27]. In addition, LRR-RLKs may participate in other biological processes such as plant growth, development and response to abiotic stresses [28]. For example, CLAVATA1 (CLV1) determines shoot and floral meristem size in *Arabidopsis* [29]. *OsGIRL1*-overexpressing transgenic *Arabidopsis* plants were hypersensitive in response to salt stress and heat stress, or hyposensitive in response to gamma-ray treatment and osmotic stress [30]. However, the majority members within LRR-RLKs family in different plant species remain functionally unidentified. In particular, so far, no LRR-RLK has been functionally characterized during pepper response to *R. solanacearum.*

A key step in both PTI and ETI is the massive transcriptional reprogramming dictated by various transcription factors. Through integrating the signal initiation and along the signaling pathways transmitting them into appropriate transcription of many defense associated genes including PRRs, R proteins and other signaling components [31–33], transcription factors play important roles in the regulation of plant immunity [34–38]. The HD-Zip transcription factors, characterized by two conserved domains, the DNA-binding homeodomain (HD) and an adjacent leucine zipper (LZ) motif, are unique to plants [39]. HD-Zip proteins are classified into four subfamilies (I–IV) based on their conserved HD-Zip domain, gene structure and additional conserved motifs [40]. HD-Zip I members were reported to play essential roles in response to environmental conditions [41, 42]. A HD-Zip I protein in pepper, CaHDZ27, was previously found to function as a positive regulator in plant immunity toward *R. solanacearum* by forming homodimers [42]. However, how CaHDZ27 linked with the upstream signaling components remains to be elucidated.

In the present study, a pepper putative LRR-RLK gene *CaLRR-RLK1* which is up-regulated by the inoculation of *R. solanacearum* was functionally characterized in immunity, and its transcriptional regulation by CaHDZ27 was identified. The results showed that *CaLRR-RLK1* act as a positive regulator in pepper response to RSI, the promoter of *CaLRR-RLK1* is targeted by CaHDZ27 in a pseudopalindromic DNA (CAATTATTG) dependent manner, and it is up-regulated by CaHDZ27 during pepper response to RSI.

## Results

### Isolation and sequence analysis of *CaLRR-RLK1*

**Fig. 1:**
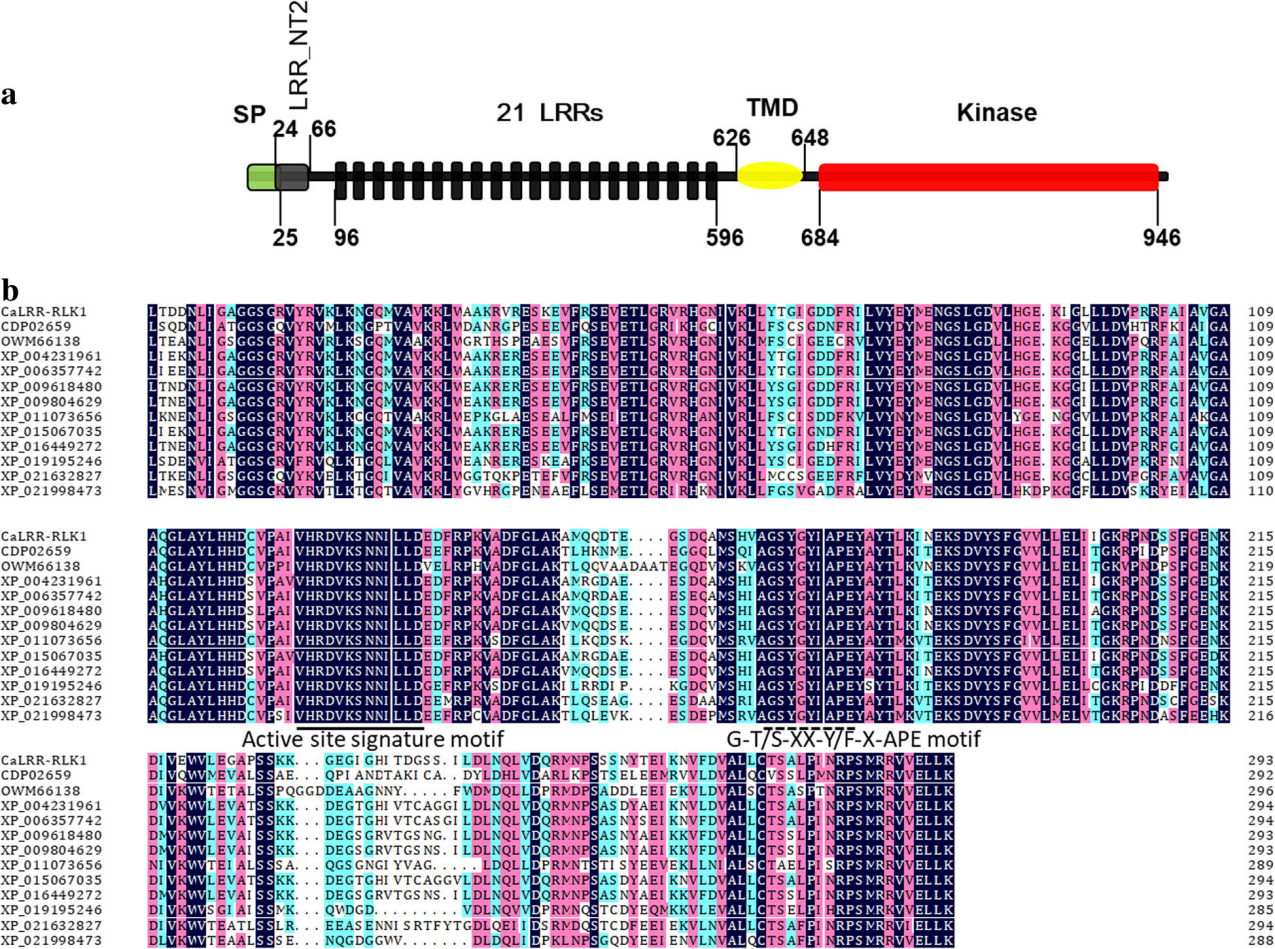
The deduced amino acid sequence analysis of CaLRR-RLK1. **a** Schematic diagram of CaLRR-RLK1 protein domain architecture. **b** Comparison of the protein kinase subdomain of CaLRR-RLK1with that of serine/threonine protein kinases from *Ipomoea nil* (XP_019195246), *Manihot esculenta* (XP_021632827), *Punica granatum* (OWM66138), *Sesamum indicum* (XP_011073656), *Helianthus annuus* (XP_021998473), *Solanum lycopersicum* (XP_004231961), *Solanum tuberosum* (XP_006357742), *Solanum pennellii* (XP_015067035), *Nicotiana sylvestris* (XP_009804629), *Nicotiana tabacum* (XP_016449272), *Nicotiana tomentosiformis* (XP_009618480) and *Coffea canephora* (CDP02659)

A putative LRR-RLK gene in pepper was originally identified as up-regulation in pepper plants challenged by *R. solanacearum* in our previous gene differential expression display by cDNA-AFLP (cDNA-amplified fragment length polymorphism). For cDNA-AFLP analysis, 64 primer pairs were used for selective PCR amplification and total 400 transcript derived fragments (TDFs) obtained. Among them, 114 displayed altered expression patterns after inoculation of *R. solanacearum*, of which 35 up-regulated and 79 down-regulated. The corresponding gene sequence including the promoter, 5’UTR, 3’UTR and the CDS were identified using pepper genome sequence (http://peppersequence.genomics.cn/page/species/index.jsp). The gene product was designated *CaLRR-RLK1* (GenBank accession number: XP_016560889) as it represents the first report of a LRR-RLK from pepper. The full-length complementary DNA (cDNA) sequence of *CaLRR-RLK1* was cloned by PCR with a specific pair of primers. By sequence analysis, the cDNA contains an open reading frame (ORF) of 2955 bps in length, its deduced amino acid sequence are 984 aa in length, containing a predicted extracellular region with a N-terminal signal peptide (SP, residues 1–25), a LRR_NT2 (a leucine-rich repeat N-terminal domain (residues 24–66), 21 extracellular leucine-rich repeats (LRRs, residues 96–596), a trans-membrane domain (TMD, residues 626–648) and a kinase domain (residues 684–946) (Fig. 1a).

The kinase domain shares high sequence homology with that of other RLK proteins (Fig. 1b). The conserved active site signature motif (VHRDVKSNNILLD residues) and the G-T/S-XX-Y/F-X-APE motif [43] indicate that CaLRR-RLK1 is a Ser/Thr kinase rather than a Tyr kinase. In addition, CaLRR-RLK1 appear to be a RD type protein kinase since it contains a conserved arginine-aspartic acid motif in its kinase subdomain [4].

Phylogenetic tree analysis was performed based on the amino acid sequence of CaLRR-RLK1 and its close homologs in other plant species. The result showed that CaLRR-RLK1 falls into a clade with *Solanum tuberosum* LRR-RLK (XP_006357742), *Solanum lycopersicum* LRR-RLK (XP_004231961) and *Solanum pennellii* LRR-RLK (XP_015067035) (Additional file 1: Figure S1).

### Expression of *CaLRR-RLK1* induced by *R. solanacearum* or exogenous hormones

**Fig. 2:**
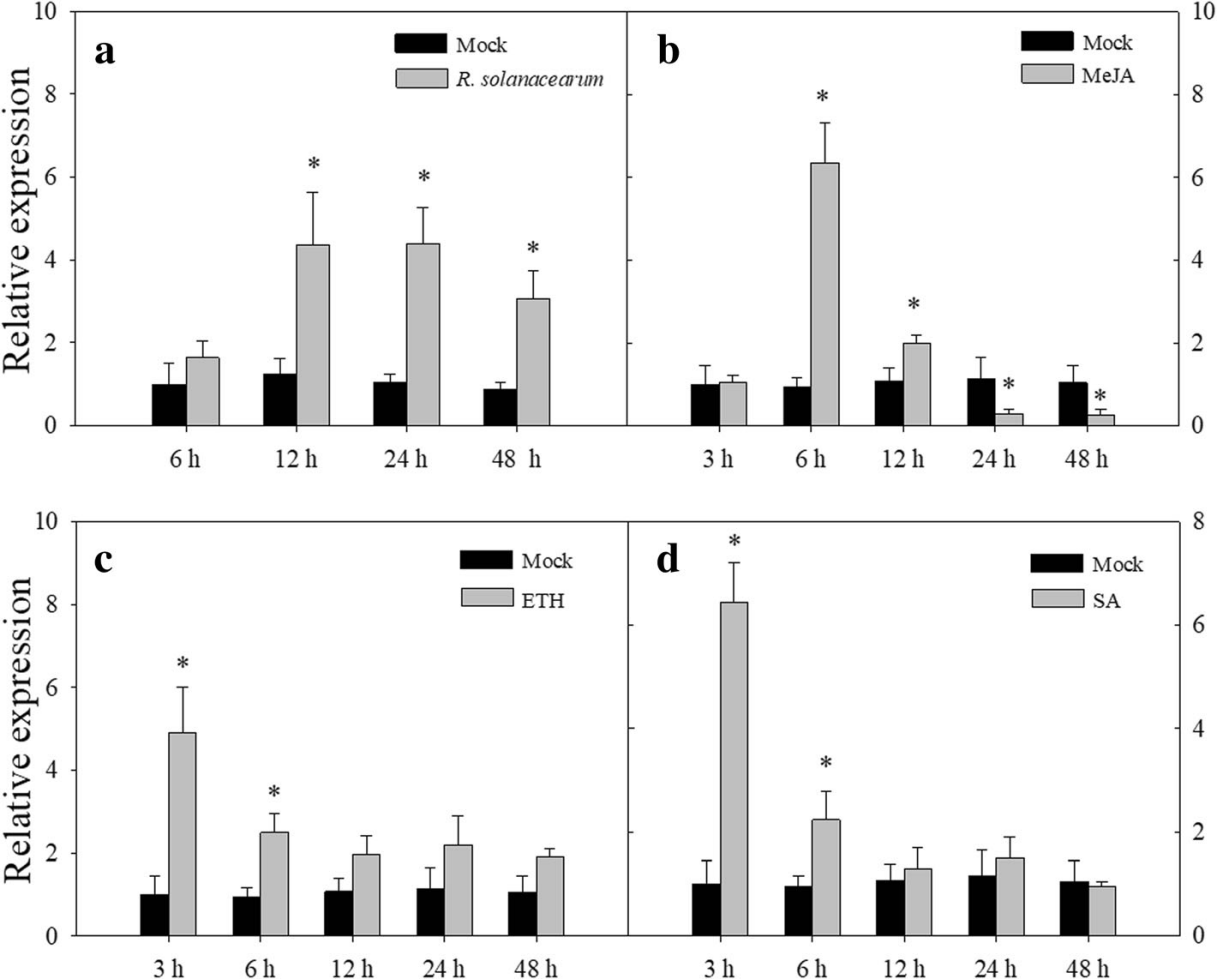
The transcription of *CaLRR-RLK1* in pepper leaves inoculated with *R. solanacearum* as well as exogenous application of methyl jasmonate (MeJA), ethephon (ETH) or salicylic acid (SA). **a** qRT–PCR analysis of transcript levels of*CaLRR-RLK1* in pepper leaves challenged with *R. solanacearum* inoculation compared to the mock treatment. **b**-**d** Relative transcript levels of *CaLRR-RLK1* gene in leaves of pepper plants after treated with SA, MeJA or ETH. Values are the mean ± standard error (*n* = 6).Asterisks (*P* < 0.05) indicate significant differences as analyzed by the Student’s *t*-test

To confirm the inducible expression of *CaLRR-RLK1* in pepper plants against *R. solanacearum* inoculation***,*** we detected its transcript levels in pepper leaves by qRT-PCR analysis. The result showed that the transcript levels of *CaLRR-RLK1* were increased at 12 to 48 h post inoculation of *R. solanacearum* compared to the mock treatment (Fig. 2a)*.*

SA, JA and ET are signaling molecules that participate in the regulation of plant immunity [44–47]. To confirm the result that *CaLRR-RLK1*was induced by RSI, the transcript levels of *CaLRR-RLK1* in pepper plants treated with exogenous applications of SA, MeJA or ETH were measured by qRT-PCR. The result showed that the transcript levels of *CaLRR-RLK1* were up-regulated dramatically and peaked at 6 h post application (hpa) of exogenous application of MeJA (Fig. 2b), followed by a gradual decrease during 12–48 hpa. Upon application of ETH, the *CaLRR-RLK1* transcript abundance was induced as early as 3 hpa (Fig. 2c). Similarly, the maximum transcripts occurred at 3 hpa of SA and then decreased to basal levels at 12 hpa (Fig. 2d). These results imply that *CaLRR-RLK1* is involved in pepper defense against *R. solanacearum* regulated by a signaling mediated by SA, JA and ET.

### Subcellular localization of CaLRR-RLK1

**Fig. 3:**
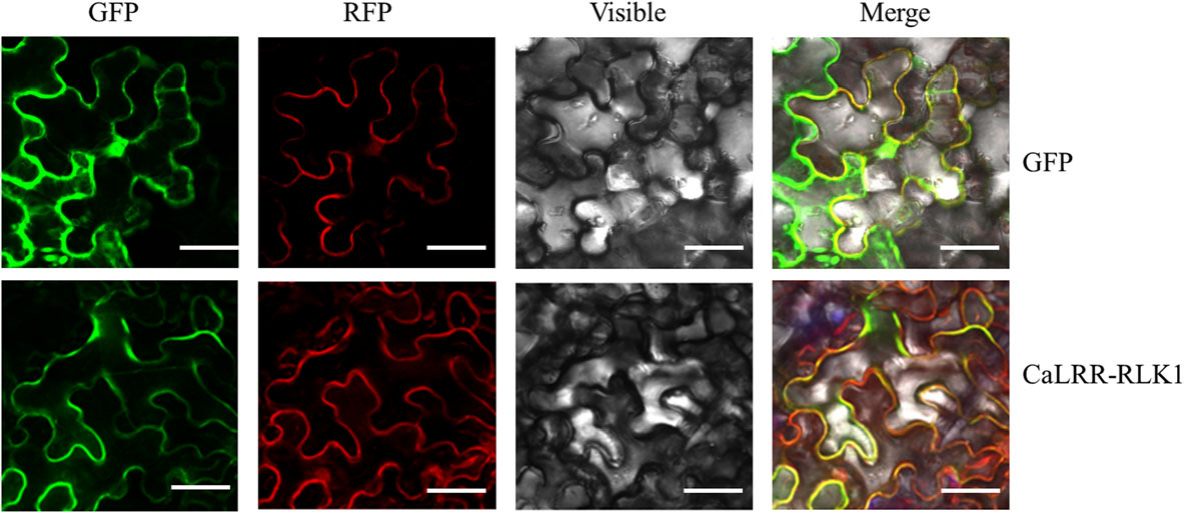
Subcellular localization of CaLRR-RLK1 by agro-infiltration in mesophyll cells of *Nicotiana benthamiana* leaves. Transient co-overexpression of CBL1n-RFP andCaLRR-RLK1-GFP or GFP in *N. benthamiana* leaves. Fluorescencewas detected using a confocal microscope. GFP, green fluorescent protein; RFP, red fluorescent protein. Bars = 50 μm

To study the subcellular localization of CaLRR-RLK1, we fused the full length ORF of CaLRR-RLK1 without the termination codon to 5′ terminal of the green fluorescent protein (GFP), resulting *35S*:*CaLRR-RLK1-GFP* construct, using *35S*:*GFP* and *35S*:*CBL1n-RFP* (specifically targeting to the plasma membrane) [48] as controls. By agro-infiltration, *CaLRR-RLK1-GFP* and *CBL1n-RFP* or *GFP* and *CBL1n-RFP* were transiently co-overexpressed in *Nicotiana benthamiana* leaves. The results showed that green fluorescent signal of CaLRR-RLK1-GFP completely overlapped with the red fluorescent signal in the plasma membrane, while the control GFP was ubiquitously distributed throughout the cell (Fig. 3), indicating the localization of CaLRR-RLK1 in the plasma membrane.

### *CaLRR-RLK1* silencing in pepper results in enhanced susceptibility to *R. solanacearum*

**Fig. 4:**
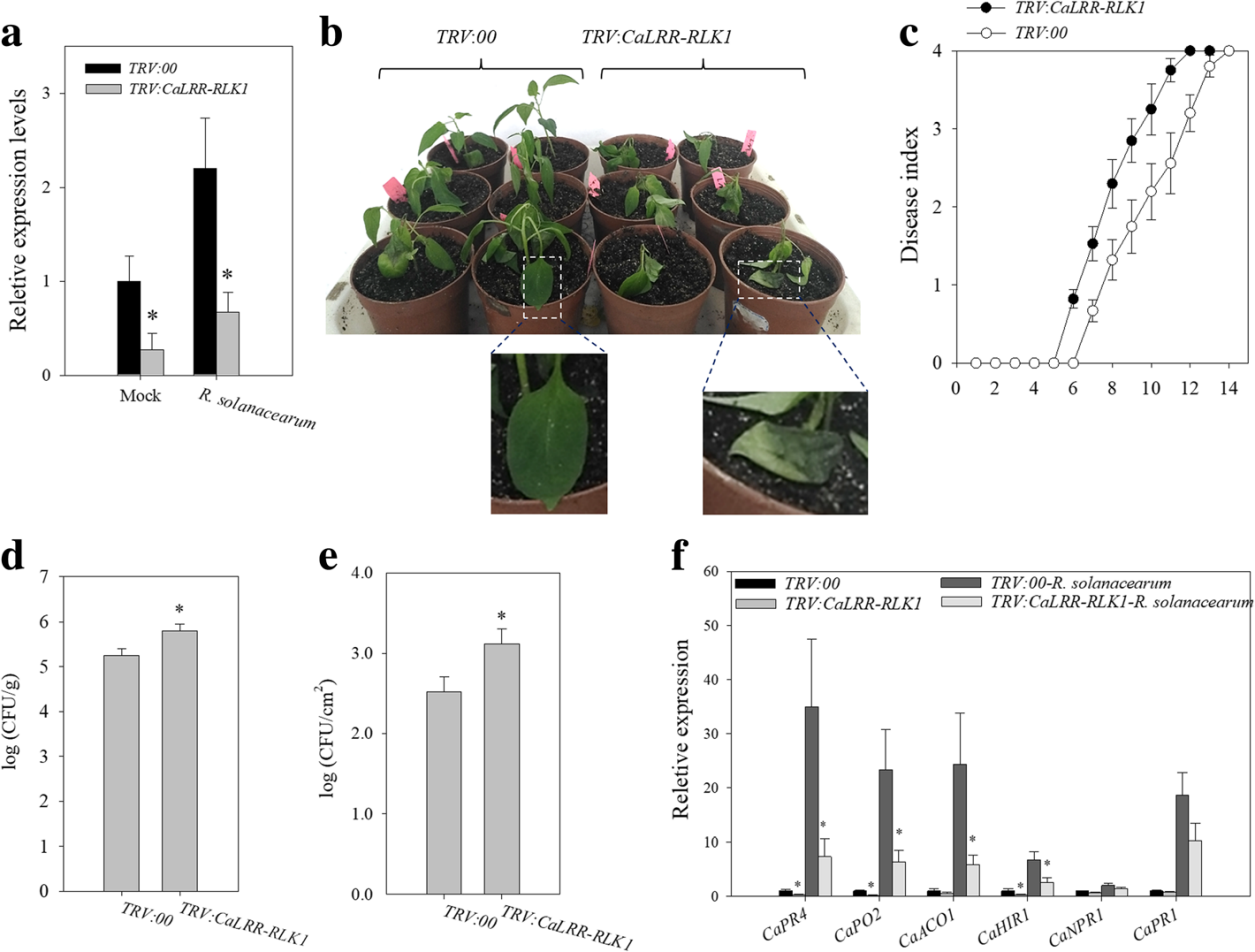
Silencing of *CaLRR-RLK1*enhanced susceptibility of pepper plants to RSI. **a** The efficiency of *CaLRR-RLK1* by VIGS was detected by qRT-PCR in *TRV*:*CaLRR-RLK1* and *TRV*:*00* plants challenged by *R.solanacearum.* **b** Disease symptoms of pepper plants at 10 dpi with *R. solanacearum*. **c** Disease index of *R. solanacearum* were evaluated daily for 14 days using a scale of 0 to 4. At least 50 plants were inoculated with *R. solanacearum* by root irrigation for *TRV*:*CaLRR-RLK1* or *TRV*:*00* plants. Results shown are the means of three independent experiments, each with 10 plants for *TRV*:*CaLRR-RLK1* or*TRV*:*00*,bars indicate standard error of the mean. **d** Growth of *R. solanacearum* in stems of *TRV:CaLRR-RLK1* and *TRV*:*00* plants at 9 days post inoculation of *R. solanacearum* by root irrigation. **e** Growth of *R. solanacearum* in leaves of *TRV:CaLRR-RLK1* and *TRV*:*00* plants at 3 days post leaf-inoculation of *R. solanacearum*. **f** The relative transcript levels of the defense related genes in leaves of *TRV*:*CaLRR-RLK1*and*TRV*:*00* plants. In **a**, **d**, **e** and **f**, values were the mean ± standard error (n = 6). Asterisks (*P* < 0.05) indicate significant differences as analyzed by the Student’s *t*-test

To further analyze the possible role of *CaLRR-RLK1* in pepper response to RSI, virus-induced gene silencing (VIGS) approach was used to investigate the effect of silencing of *CaLRR-RLK1* on pepper resistance to RSI. A fragment 182 bps in length within the 3’-UTR region of *CaLRR-RLK1* was cloned into pTRV2 vector resulting in *TRV*:*CaLRR-RLK1* (using *TRV*:*00* as a control). The *Agrobacterium* GV3101 cells harboring *TRV*:*CaLRR-RLK1* were infiltrated onto the cotyledon of the 12–14 days old pepper plants, and the *TRV*:*CaPDS* pepper plants were used to monitor the gene silencing process. When the seedlings were grown for about 25 days, plants infiltrated with *TRV*:*CaPDS* exhibited bleaching phenotype in their newly emerging leaves, the *TRV*:*00* and *TRV*:*CaLRR-RLK1* pepper plants were inoculated with *R. solanacearum,* and their transcript levels of *CaLRR-RLK1* were measured by qRT-PCR using randomly selected 6 *TRV*:*CaLRR-RLK1* pepper plants to detect the silencing efficiency of *CaLRR-RLK1*. The result showed that transcript levels of *TRV*:*CaLRR-RLK1* in *R. solanacearum* inoculated or mock treated plants were about 20–30% of that in the control (*TRV*:*00*) plants (Fig. 4a), indicating the success of *CaLRR-RLK1* silencing in pepper plants by VIGS*.*

With the *CaLRR-RLK1* silencing pepper plants, which were confirmed by qRT-PCR individually, the effect of *CaLRR-RLK1* silencing on pepper immunity against RSI was assayed. All of *CaLRR-RLK1* silenced pepper plants did not exhibit any morphological changes compared to the control plants. Inoculated with *R. solanacearum* by root irrigation, *TRV*:*CaLRR-RLK1* plants exhibited significantly enhanced susceptibility to RSI compared to the control plants (Fig. 4b). After 6 days post inoculation (dpi), *TRV*:*CaLRR-RLK1* plants displayed a higher disease index than the control. *TRV*:*CaLRR-RLK1* plants began to display the bacterial wilt symptom at 6 dpi and complete wilt phenotype at 12 dpi, while the control plants did not begin to display the bacterial wilt symptom until 7 dpi and complete wilt until 14 dpi (Fig. 4c). Consistently, a higher population density of *R. solanacearum* was detected in stems of *TRV*:*CaLRR-RLK1* plants at 9 dpi by root irrigation (Fig. 4d) and also in *R. solanacearum* inoculated leaves at 3 dpi (Fig. 4e), compared to that in the control plants.

To further investigate whether silencing of *CaLRR-RLK1* affect the expression of immunity-related genes in pepper, qRT–PCR analysis was performed. The results showed that expressions of HR related *CaHIR1* [49], ROS detoxification-associated *CaPO2* [50], ET-responsive *ACC* oxidase gene *CaACO1* [51], JA/ET signaling associated *CaPR4* [52] were significantly down-regulated in *CaLRR-RLK1* silenced leaves, compared with that in the control plants challenged with RSI. However, there was no significant difference for the transcript levels of SA signaling associated *CaPR1* [53] and *CaNPR1* [54] (Fig. 4f). These results indicate that silencing of *CaLRR-RLK1* increased susceptibility of pepper plants to RSI*.*

### Transit expression of *CaLRR-RLK1* triggered HR cell death and induced the transcription of immunity associated genes

**Fig. 5:**
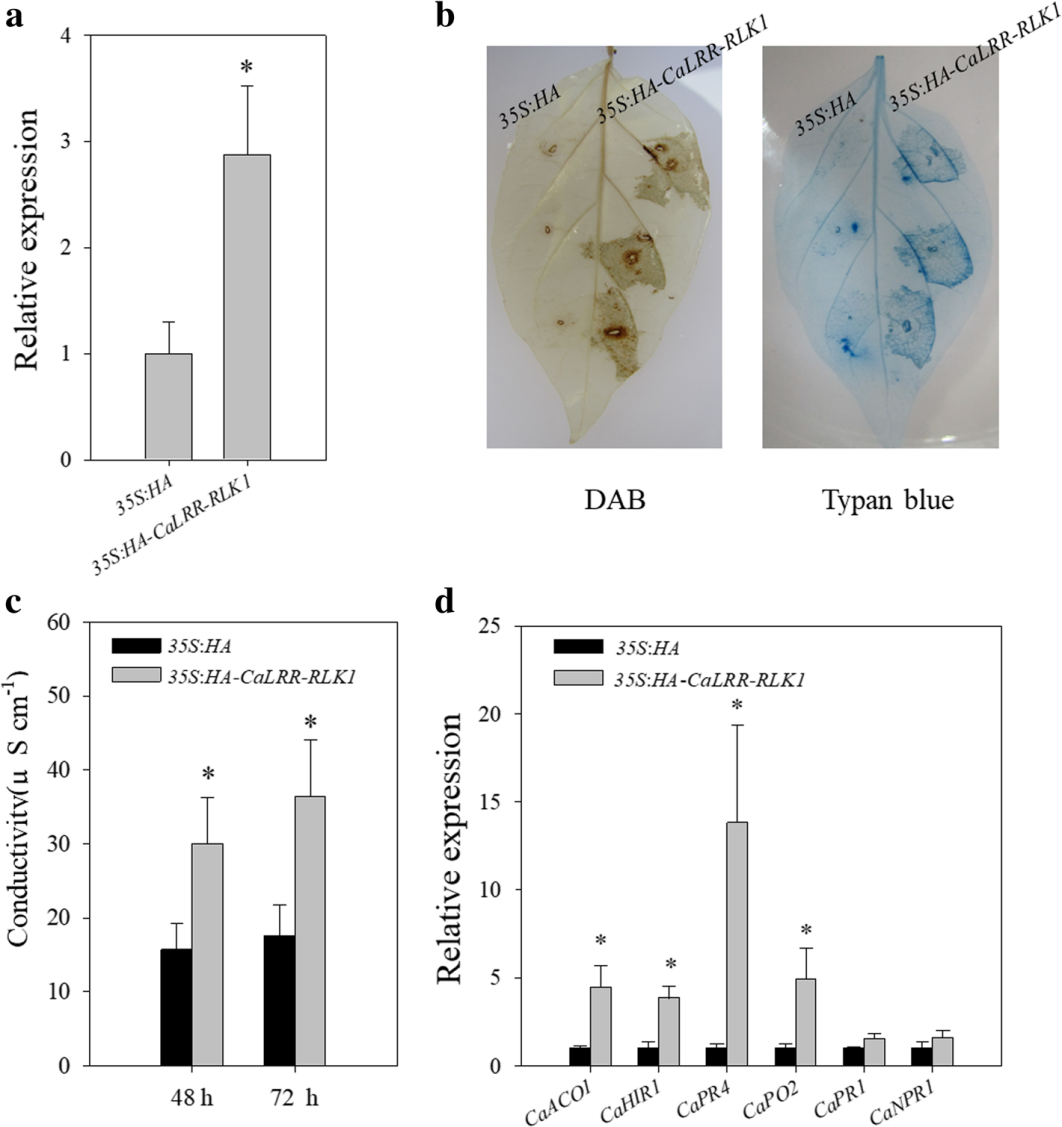
Transient overexpression of *CaLRR-RLK1*triggered HR mimic cell death in pepper leaves. **a** The transcript levels of*CaLRR-RLK1* in pepper leaves detected by qRT-PCR. **b** Trypan blue and DAB staining in leaves transiently expressing *CaLRR-RLK1* at 48 h post infiltration. **c** Electrolyte leakage from leaf discs of *CaLRR-RLK1* transiently overexpressed pepper leaves. **d** Transcript levels of immunity associated genes were detected in *CaLRR-RLK1* transient overexpression pepper leaves by qRT-PCR. In **a**, **c** and **d**, values were the mean ± standard error (n = 6). Asterisks (*P* < 0.05) indicate significant differences as analyzed by the Student’s *t*-test

To confirm the results in the gene silencing by VIGS, *CaLRR-RLK1* was transiently overexpressed via agro-infiltration. GV3101 cells containing 35S:*HA-CaLRR-RLK1* or 35S:*HA* were infiltrated into pepper leaves, the success of the transient overexpression was confirmed by qRT-PCR (Fig. 5a) or immunoblot with antibodies of HA (Additional file 2: Figure S2). Darker staining of trypan blue and DAB were detected in *CaLRR-RLK1* transiently overexpressing pepper leaves around the infiltration sites, while no such darker staining was detected in the control plant leaves (35S:*HA*), indicating that HR cell death and H2O2 accumulation were triggered by transient overexpression of *CaLRR-RLK1* (Fig. 5b)*.* Consistently, a significant enhanced electrolyte leakage was also detected in *CaLRR-RLK1* transiently overexpressing pepper leaves at 48 and 72 h post infiltration (hpi) compared with that of the control (Fig. 5c).

Quantitative RT-PCR analysis was used to investigate whether transient expression of *CaLRR-RLK1* affect defense associated genes including *CaACO*1, *CaHIR1*, *CaPR4*, *CaPO2, CaNPR1* and *CaPR1* in pepper leaves. The results showed that *CaACO*1, *CaHIR1*, *CaPR4* and *CaPO2* exhibited enhanced transcript levels in *CaLRR-RLK1* transiently overexpressing pepper leaves at 48 hpi compared to that in the control, transcript levels of *CaNPR1* and *CaPR1* did not alter (Fig. 5d).

### Ectopic overexpression of *CaLRR-RLK1* enhanced resistance of tobacco plants to RSI

To further confirm that result that CaLRR-RLK1 act as positive regulator in plant immunity, we investigated the effect of its ectopic overexpression on the resistance of tobacco plants to RSI by developing the transgenic tobacco lines via *Agrobacterium* mediated method. Six transgenic lines (T_3_) each with at least 10 seedlings were confirmed by kanamycin resistance analysis. Two lines were employed to assay the transcriptional expression of *CaLRR-RLK1,* the seedlings were inoculated with *R. solanacearum* cells. RT-PCR result showed that *CaLRR-RLK1* constitutively expressed in leaves of the two lines (#2 and #8) (Additional file 3: Figure S3).

**Fig. 6:**
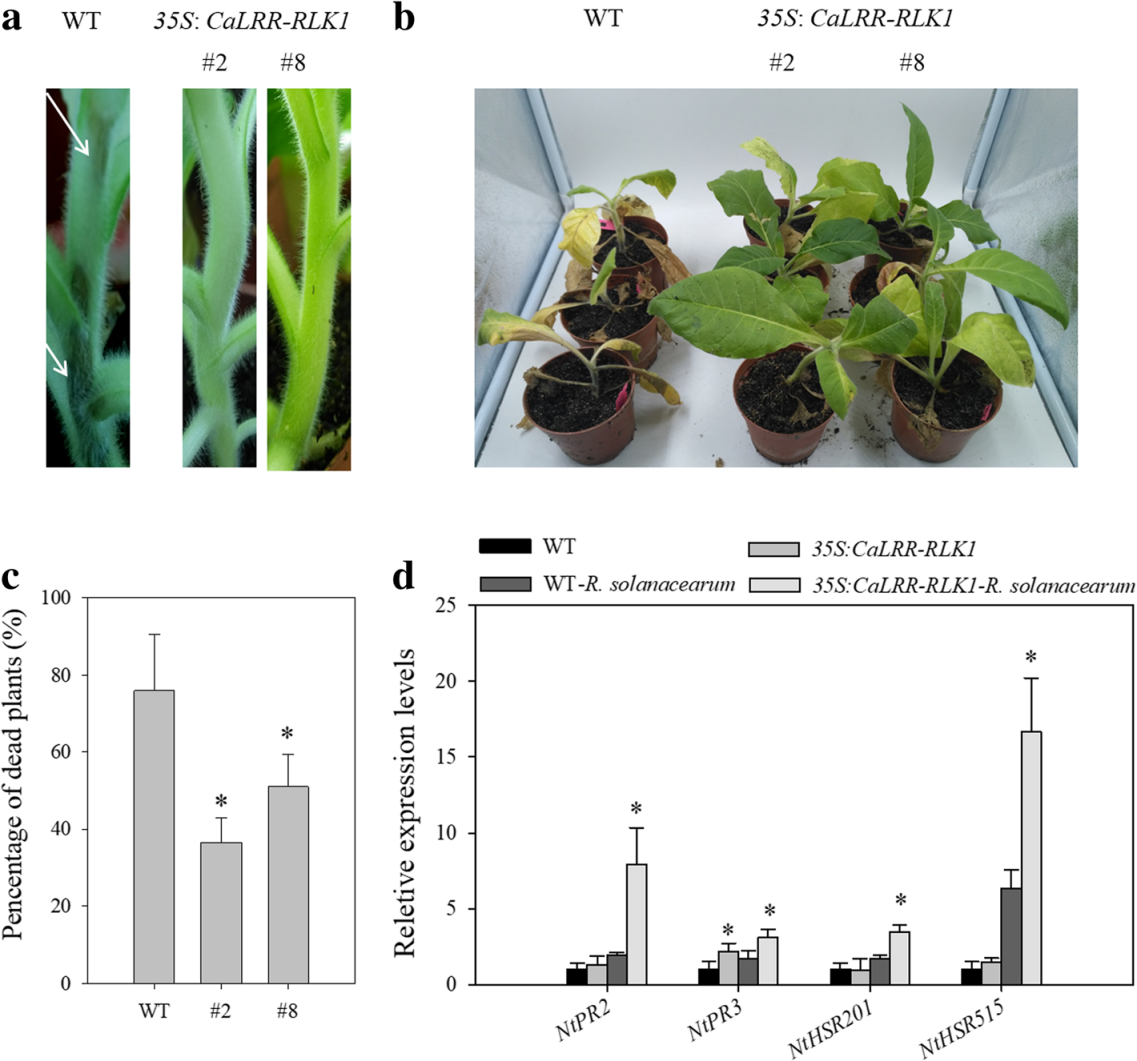
Ectopic overexpression of *CaLRR-RLK1*enhanced resistance of tobacco plants to RSI. **a** Comparison of the symptom on stems between wild-type and *CaLRR-RLK1* overexpressing lines at 7 dpi with *R. solanacearum.* **b** Comparison of wilt symptoms in seedlings of *CaLRR-RLK1* overexpressing lines and the wild-type at 21 dpi. **c** Percentage of dead plants at 28 dpi with *R. solanacearum* by root irrigation. Data were from at least 10 plants for each line. Three independent experiments were performed. Bars represent standard error of the mean. **d** Relative transcript levels of immunity associated genes in plants of *CaLRR-RLK1* overexpressing lines and wild-type at 48 h post inoculated with *R. solanacearum*. Values were the mean ± standard error (*n* = 3). Asterisks (*P* < 0.05) indicate significant differences as analyzed by the Student’s *t*-test

Eight-week-old tobacco plants were inoculated with *R. solanacearum* by root irrigation, the development of bacterial wilt symptoms was monitored. At 7 dpi, wilting and contagion symptoms occurred on the stems of the wild-type plants, while no significant symptom was observed in plants of *CaLRR-RLK1* overexpressing lines (Fig. 6a). At 21 dpi, wild-type plants exhibited extremely severe wilting symptoms, but only slight wilt symptoms were observed in plants of *CaLRR-RLK1* overexpressing lines (Fig. 6b). At 28 dpi, a significant higher death rate (76.1%) was found in the wild-type plants than that in plants of the two lines, which was 36.5 and 51.1%, respectively (Fig. 6c).

In addition, the transcript levels of immunity associated genes, including SA-induced pathogenesis-related *NtPR2* [55] and *NtPR3* [56], HR-related *NtHSR201* and *NtHSR515* [57], were measured by qRT-PCR in tobacco plants of *CaLRR-RLK1* overexpressing lines and the wild type, the results showed that the transcript levels of *NtPR2*, *NtPR3*, *NtHSR201* and *NtHSR515* increased in transgenic plants compared to that in wild-type plants in response to *R. solanacearum* infection (Fig. 6d).

### The promoter of *CaLRR-RLK1* was directly bound and transcriptionally modulated by CaHDZ27

**Fig. 7:**
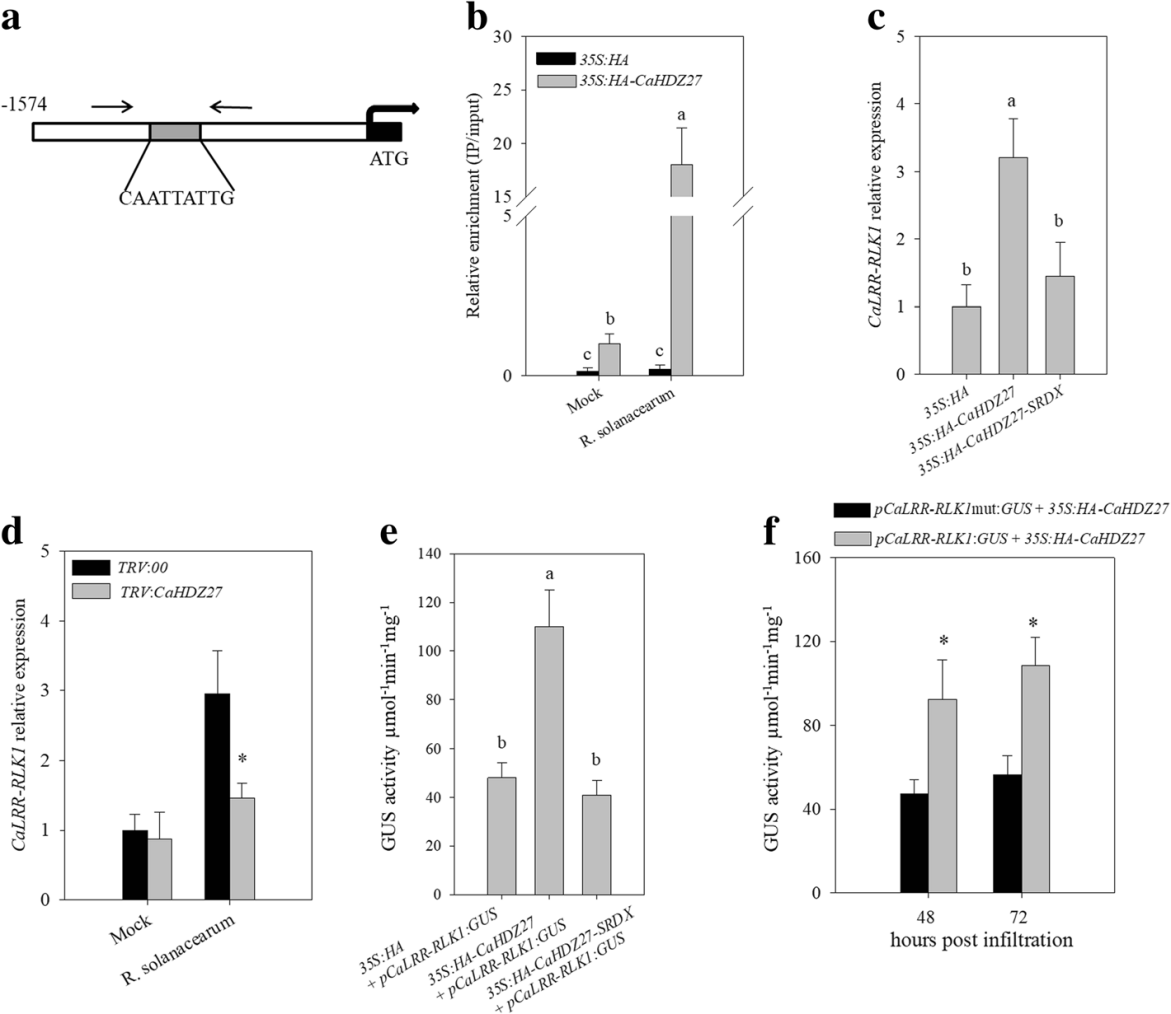
CaHDZ27 can bind the motif CAATTATTG in the promoter of*CaLRR-RLK1* andactivate*CaLRR-RLK1* transcription. **a** Scheme of the *CaLRR-RLK1* promoter region, primers used in ChIP-qPCR assay were marked by arrows. **b** ChIP-qPCR assays were performed using the specific primers corresponding to the region containing the motif (CAATTATTG). **c** The transcript levels of *CaLRR-RLK1* in *HA-CaHDZ27-SRDX* or *HA-CaHDZ27* transiently overexpressed pepper leaves. **d** The transcript levels of *CaLRR-RLK1* in *CaHDZ2*7-silenced leaves challenged by *R. solanacearum*. **e** GUS activities driven by the promoter of *CaLRR-RLK1* were assayed when transiently overexpressing *HA-CaHDZ27-SRDX* or *HA-CaHDZ27* in pepper leaves. **f** Activities of GUS driven by the p*CaLRR-RLK1* or *pCaLRR-RLK1mut,*in which CAATAATTG was replaced by CA**GGGG**TTG, when co-transiently overexpressed with*HA-CaHDZ27* in pepper leaves. In **b**, **c** and **e**, the values were the mean ± standard error (n = 3), different letters indicate significant differences, as determined by LSD test (*P* < 0.05). In **d** and **f**, asterisks indicate significant difference according to Student’s *t* test at *P* < 0.05

Silencing of *CaLRR-RLK1* impairs resistance of pepper to RSI, while its overexpression enhances the resistance of pepper or tobacco to RSI. Therefore, its inducible expression under challenge by RSI is crucial for its role as positive regulator. To study the molecular mechanism underlying this inducible expression, the *cis*-elements in the promoter region of *CaLRR-RLK1* of 1574 bps in length was analyzed (http://bioinformatics.psb.ugent.be/webtools/plantcare/html/). Noticeably, a 9 bp pseudopalindromic DNA sequence (CAATTATTG), which is potentially bound by HD-Zip transcription factors [42, 58], was found to be present in the promoter (Fig. 7a). Since our previous study found that CaHDZ27, a HD-Zip I transcription factor, acts a positive regulator in pepper response to RSI, we speculated that CaHDZ27 might act as an upstream regulator of *CaLRR-RLK1* by binding the motif CAATTATTG in its promoter. To test if it is the case, we employed chromatin immunoprecipitation (ChIP) assay via *HA-CaHDZ27* transient overexpression in pepper leaves, the chromatins were isolated from *HA-CaHDZ27* transiently overexpressing pepper leaves after crosslinking with 1% formaldehyde, sheared into fragments of 300-500 bp in length, the DNA fragments bound to CaHDZ27 were immunoprecipitated by antibodies of HA, the de-crosslinked and purified DNA fragments were used as template for qRT- PCR with specific primer pairs based on the sequence flanking the motif (CAATTATTG) in the promoter of *CaLRR-RLK1*, the result showed that the promoter of *CaLRR-RLK1* was significantly enriched by CaHDZ27, and in response to the challenge of *R. solanacearum*, CaHDZ27 binding enrichment was markedly increased (Fig. 7b), indicating that CaHDZ27 binds to the promoter of *CaLRR-RLK1* probably via the motif CAATTATTG.

To assay if *CaLRR-RLK1* is transcriptionally modulated by CaHDZ27, we carried out transient overexpression of *HA-CaHDZ27* in pepper leaves by infiltration with GV3101 cells containing *35*:*HA-CaHDZ27* or *35S*:*HA*, 2 days after agro-infiltration, the transcript levels of *CaLRR-RLK1* were checked by qRT-PCR. The result showed that *CaLRR-RLK1* was up-regulated in leaves infiltrated with *35S:HA-CaHDZ27*, and not down-regulated in leaves infiltrated with *35S*:*HA-CaHDZ27-SRDX* (a repressor version of *HA-CaHDZ27)*, compared with *35S:HA* (Fig. 7c). Moreover, we silenced *CaHDZ27* in pepper plants by VIGS and checked the transcript level of *CaLRR-RLK1* in *CaHDZ27-*silenced plants by qRT-PCR, the result showed that the transcript level of *CaLRR-RLK1* decreased in *TRV*:*CaHDZ2*7 pepper leaves upon *R. solanacearum* inoculation compared to that in the control (*TRV*:*00*) plants (Fig. 7d)*.* To provide additional evidence that the transcript of *CaLRR-RLK1* was activated by CaHDZ27, the promoter of *CaLRR-RLK1* was cloned and inserted into pMDC163 [59] to get construct *pCaLRR-RLK1*:*GUS*. The GV3101 cells containing *pCaLRR-RLK1*:*GUS* were mixed with *35S:HA-CaHDZ27*, *35S:HA-CaHDZ27-SRDX* or *35S:HA* at 1:1 ratio, and infiltrated into the pepper leaves. GUS activity was measured at 2 dpi, the result showed that GUS activities were enhanced by *HA-CaHDZ27* transient overexpression, while not decreased by *HA-CaHDZ27-SRDX* compared to that in the control leaves (Fig. 7e), consistently with the qRT-PCR results. These results indicated that *CaLRR-RLK1* is transcriptionally activated by CaHDZ27*.*

To further determine whether the transcriptional activation of *CaLRR-RLK1* by CaHDZ27 is via the motif CAATTATTG within the promoter of *CaHDZ27*, we mutated the *cis*-acting element (CAGGGGTTG) in the promoter region of *CaLRR-RLK1*, and resulted in construct *pCaLRR-RLK1mut*:*GUS.* GV3101 cells containing *pCaLRR-RLK1*:*GUS* or *pCaLRR-RLK1mut:GUS* were mixed with *35S*:*HA-CaHDZ27* and were co-infiltrated into the leaves of *N. benthamiana*. 48 or 72 h after infiltration, the GUS activity was measured. The result showed that GUS activity was much higher in the leaves co-expressing *pCaLRR-RLK1*:*GUS* and *35S*:*HA-CaHDZ27* than that in *pCaLRR-RLK1mut*:*GUS* and *35S*:*HA-CaHDZ27* co-expressed pepper leaves (Fig. 7f). Taken together, these data support the idea that CaHDZ27 activates the transcription of *CaLRR-RLK1* by direct binding to the CAATTATTG motif.

## Discussion

### CaLRR-RLK1 plays a positive role in pepper immunity

Bacterial wilt incited by *R. solanacearum* causes heavy losses in pepper, a vegetable of great agricultural importance worldwide. In the present study, our data indicate that *CaLRR-RLK1* act as a positive regulator in pepper immunity toward *R. solanacearum* and it is transcriptionally modulated directly by CaHDZ27.

Based on sequence motifs in the extracellular domain, typical RLKs can be divided into more than 21 subfamilies [11]. So far, three RLKs including CaRLK1 [60–62], CaPIK11 [63–66] and CaLecRK-S.5 [67] in pepper have been functionally characterized. Like CaLRR-RLK1 in the present study, CaRLK1 contains cytoplasmic kinase domain, a transmembrane span, and an extracellular domain which shows low homology to other known plant RLKs. It functions in cell death [62], hypoxia resistance [61] and cell division [60]. CaPIK1 functions as a positive regulator of defense responses and cell death in an SA-dependent manner [65, 66]. L-type lectin receptor kinase CaLecRK-S.5 positively mediated broad-spectrum resistance in pepper [67]. However, no LRR-RLK in pepper has been functionally characterized in plant immunity to date.

CaLRR-RLK1 contains a predicted extracellular region with an N-terminal signal peptide, 21 extracellular leucine-rich repeats, a trans-membrane domain and an intracellular kinase domain. CaLRR-RLK1 appears to be RD Ser/Thr kinase since it contains a conserved arginine-aspartic acid motif in its kinase subdomain. Consistent with the trans-membrane domain, CaLRR-RLK1 exclusively localizes to the plasma membrane. The direct evidence that CaLRR-RLK1 act as a positive regulator came from data that *CaLRR-RLK1* silencing by VIGS significantly impaired resistance of pepper plant to RSI and down-regulated the tested immunity associated maker genes. By contrast, the transient overexpression of *CaLRR-RLK1* significantly enhanced the HR cell death which is frequently found to occur in ETI and occasionally in PTI [68], the accumulation of H2O2 which is manifested by DAB staining and closely related to HR cell death [69], and the induction of the tested immunity associated marker genes. These data from pepper plants indicate a role of CaLRR-RLK1 as a positive regulator in pepper immunity against RSI, and it was further supported by the data from ectopic expression of CaLRR-RLK1 in tobacco plants. The overexpression of *CaLRR-RLK1* significantly enhanced the resistance of tobacco plants to RSI, accompanied with significant up-regulations of the tested immunity associated marker genes including *NtPR2* [55], NtPR3 [56], *NtHSR201* and *NtHSR515* [57].

RLKs were categorized into RD kinases and non-RD kinases according to the RD motif in their kinase domains [70]. By far, well-studied LRR-RLK PRRs, such as FLS2 or Xa21 [71, 72], that recognize the exogenous PAMPs in disease resistance were non-RD kinases [70]. Characterized plant DAMP receptors all contained RD kinase domains [4]. For example, PEPR1/2 and RLK7 have been implicated in activation of PTI against pathogens through perception of peptides AtPEP1 [73, 74] and PIP1 [75] in *Arabidopsis*, respectively. As a RD kinase domain containing RLK, we speculate that CaLRR-RLK1 might act as a receptor of unidentified DAMPs in pepper defense response to *R. solanacearum*.

### The expression of *CaLRR-RLK1* was activated by CaHDZ27 direct binding the CAATTATTG motif in its promoter

Our data indicate an inducible expression of *CaLRR-RLK1* upon challenge of *R. solanacearum,* this enhanced expression might be crucial for profound amplification of the defense signaling to avoid the negative effect of the constitutive immune reaction. Some PRRs such as *FLS2* [76] and R genes such as *Xa1* [77] were reported to exhibit inducible expression upon pathogen attack. To dissect the mechanism underlying up-regulation of *CaLRR-RLK1* upon RSI, its upstream transcription factor is identified. Our data showed that CaHDZ27, a HD-Zip protein that acts as positive regulator in pepper resistance against *R. solanacearum* [42], directly targets the promoter of *CaLRR-RLK1*, and activate the expression of *CaLRR-RLK1* in a motif CAATAATTG dependent manner. Similar to *CaHDZ27* [42], the transcription of *CaLRR-RLK1* was also up-regulated by exogenously applied SA, MeJA and ETH, indicating that CaHDZ27 and *CaLRR-RLK1* act as a positive regulators in plant immunity during pepper response to RSI. CaLRR-RLK1 contains 21 LRRs in the extracellular domain. Although the ligand of LRRs is not known, CaLRR-RLK1 may play a role in the pepper defense signaling pathway through these LRR domains binding with ligand, and the enhanced expression caused by the transcript factor CaHDZ27 is beneficial to pepper resistance against *R. solanacearum*.

## Conclusions

Our data in the present study indicate that expression of *CaLRR-RLK1* was induced by *R. solanacearum* inoculation or exogenous application of SA, MeJA or ETH. Inhibition of its expression in pepper disrupted the defense ability of plants against *R. solanacearum* infection. Transit expression of this gene in pepper leaves can lead to cell death, and overexpression of this gene rendered the transgenic tobacco plants with increased disease tolerance. Furthermore, the expression of *CaLRR-RLK1* is activated by CaHDZ27 in a pseudopalindromic DNA (CAATTATTG) dependent manner. The experimental evidence collectively demonstrates that CaLRR-RLK1 act as a positive regulator in pepper response to *R. solanacearum*, and it is transcriptionally activated by CaHDZ27.

## Methods

### Plant materials and conditions of growth

Pepper (*Capsicum annuum* cultivar L. cv. Fj8), with medium-resistance to *R. solanacearum,* was obtained from the pepper breeding group at Fujian Agriculture and Forestry University. Tobacco (*Nicotiana tabacum* L. cv. Honghuadajinyuan) was kindly supplied by the tobacco breeding group in Fujian Agriculture and Forestry University. Plants were grown under the conditions of 26 °C temperature, 60% relative humidity, 60–70 μmol photons m^− 2^ s^− 1^ and 16 h light/8 h dark.

### Pathogen inoculation

Virulent strains FJC100301 and FJ1003 strain of *R. solanacearum* were from pepper and tobacco, respectively. The pathogen strains were amplified according to the method described previously [78]. The *R. solanacearum* strain was grown in PSA medium (200 g/L of potato, 20 g/L of sucrose, 3 g/L of beef extract, 5 g/L of tryptone). Bacteria were harvested with sterile ddH_2_O and re-suspended to 1.0 × 10^8^ CFU/ mL. Seedlings were root inoculated by pouring either 30 mL bacterial inoculum or sterile ddH_2_O as the mock into each pot. Before inoculation, the roots were damaged by making holes in the soil of each pot. For leaf inoculation, the third leaf from the top of each pepper plant at the eight-leaf stage were infiltrated with 10 μL of the *R. solanacearum* suspension using a syringe without a needle, and the mock treatment was performed with sterile ddH_2_O.

### Exogenous hormones treatments

Six leaf-stage pepper plants were used to investigate gene expression. For salicylic acid (SA), methyl jasmonate (MeJA) and ethephon (ETH) treatments, 1 mM SA, 100 μM MeJA or 100 μM ETH were sprayed onto pepper leaves. In parallel experiments, corresponding solvent or sterile water was sprayed as a control. Pepper leaves were sampled at various time points, frozen and stored at − 80 °C for RNA isolation.

### Subcellular localization

The open reading frame (ORF) of *CaLRR-RLK1* without the stop codon was PCR amplified from plant cDNA and cloned into pMDC83 to yield *35S*:*CaLRR-RLK1-GFP. Agrobacterium tumefaciens* strain GV3101 containing the constructs *35S*:*CaLRR-RLK1-GFP* or *35S*:*GFP* (used as a control) was re-suspended (OD_600_ = 0.8) in the induction medium (10 mM MES, 10 mM MgCl_2_, 200 μM acetosyringone, pH 5.6) and infiltrated into leaves of *Nicotiana benthamiana.* After 2 days post infiltration, GFP fluorescence was imaged using a Laser Scanning Confocal Microscope (TCS SP8, Leica, Solms, Germany) with an excitation wavelength of 488 nm and a 505–530 nm band-pass emission filter.

### VIGS in pepper plants

TRV (tobacco rattle virus)-based VIGS analysis was performed as described previously [42]. A 182-bp gene specific 3′ untranslated region of *CaLRR-RLK1* was inserted into the TRV2 vector to yield *pTRV2*:*CaLRR-RLK1* by gateway cloning technique. TRV1 and *TRV2*:*00* (control) or *pTRV2*:*CaLRR-RLK1* in *A. tumefaciens* strain GV3101 were co-infiltrated into the fully expanded cotyledons of approximately 12–14 days old pepper plants. Then, the seedlings were incubated at 16 °C for 56 h, and grown at 26 °C.

### *Agrobacterium*-mediated transient expression in pepper leaves

The full length cDNA of *CaLRR-RLK1* or *CaHDZ27* was cloned into pEarleyGate201 to yield *35S*:*HA*-*CaLRR-RLK1* construct or *35S*:*HA*-*CaHDZ27* by gateway cloning technique. The *CaHDZ27* dominant repression construct (*35S*:*HA-CaHDZ27-SRDX*) was created by fusing the *CaHDZ27* cDNA in frame with the dominant EAR repression sequence [79], which was ligated downstream of the 35S promoter into the vector pEarleyGate201.

*A. tumefaciens* strain GV3101 harboring the above vector was infiltrated into the pepper leaves at the eight-leaf stage using a syringe without a needle. After 48 or 72 h post infiltration, injected leaves were harvested for further use.

### Histochemical staining and ion conductivity measurement

Diaminobenzidine (DAB) and trypan blue staining, as well as ion conductivity measurement assays were performed according to methods described previously [51, 78]. Bacteria-infiltrated leaves were harvested and incubated in 1 mg mL^− 1^ DAB in the dark overnight and destained with lacticacid/glycerol/ethanol (1:1:3). To visualize the cell death, the infiltrated leaves were stained with trypan blue and destained with chloral hydrate solution. Cell death was quantified by ion conductivity measurement, eight discs (11 mm in diameter) were excised and washed in sterile ddH_2_O. After incubation in 20 mL of double distilled water with gentle shaking for 3 h, ion conductivity was measured using a Mettler Toledo 326 (Mettler, Zurich, Switzerland).

### Quantitative real-time RT-PCR

For quantitative real-time PCR analysis, total RNA from plant leaves was extracted using a TRIzol Reagent (Invitrogen, Carlsbad, CA, USA). First-strand cDNA was synthesized using One Step PrimeScript TM cDNA Synthesis Kit (TaKaRa, Shigo, Japan). Real-time PCR experiments were performed according to the manufacturer’s instructions for the BIO-RAD Real-time PCR system (Foster City, CA, USA) and the SYBR Premix Ex Taq II system (TaKaRa). To normalize the transcript levels, Pepper *CaActin* (GQ339766) or 18S ribosomal RNA (EF564281) and Tobacco *NtEF1α* (D63396) or *NtActin* (U60489) expression was monitored as reference genes in each reaction. The gene-specific primers used for the quantitative real-time RT–PCR analysis are listed in Additional file 4: Table S1 and Table S2. Each gene expression was calculated from three experimental replicates to ensure reproducibility of the results.

### ChIP-qPCR analysis

ChIP-qPCR assays were performed essentially as described previously [51]. Briefly, the chromatins were isolated from the pepper leaves infiltrated with *35S*:*HA*-*CaHDZ27*. The isolated chromatins were cross-linked with 1% formaldehyde, sheared, and precipitated using anti-HA. Enrichment of DNA samples was analyzed by quantitative real-time PCR. Each ChIP value was normalized to its respective input DNA value.

### β-Glucuronidase (GUS) enzymatic assay

The *CaLRR-RLK1* promoter or its mutation was cloned into pMDC163 to yield *pCaLRR-RLK1*:*GUS* or *pCaLRR-RLK1mut*:*GUS* construct. The frozen plant material collected from leaves of transiently transformed pepper, was ground with a pestle in the extraction buffer (50 mM phosphate buffer, pH 7.0, 10 mM EDTA, 0.1% Triton X-100, 0.1% sodium lauryl sarcosine, and 10 mM β-mercaptoethanol) and centrifuged at 11000 g for 10 min at 4 °C. Soluble crude protein in the supernatant was quantified using the Bradford method [80]. Quantitative analysis of GUS activity was measured with 4-methylumbelliferyl-D-glucuronide (4-MUG) as the substrate [54].

### Plant transformation

The full length cDNA of *CaLRR-RLK1* was cloned into plant binary vector pK7WG2 to yield *35S*:*CaLRR-RLK1* construct. *Agrobacterium*-mediated transformation of tobacco (*Nicotiana tabacum* L. cv. Honghuadajinyuan) was performed. Transformants were selected on Murashige and Skoog (MS) agar plates containing 50 μg mL^− 1^ kanamycin. Successful transformation was confirmed by RT-PCR using the *CaLRR-RLK1* gene-specific primers. Among the selected transgenic plants, T2 lines #2 and #8 were used in this study.

## Additional files

Additional file 1: **Figure S1.** The phylogenetic relationship of CaLRR-RLK1 protein with other plant LRR-RLKs. An unrooted neighbor-joining tree was built by MEGA 4.0. (TIF 30 kb)

Additional file 2: **Figure S2.** Transient overexpression of HA-CaLRR-RLK1 in pepper leaves detected by immunoblot. (TIF 36 kb)

Additional file 3: **Figure S3.** The expression of *CaLRR-RLK1* in representative T_3_ transgenic tobacco plants was checked by RT-PCR. Wild-type (WT) tobacco plants as the blank control, and *NtEF1α* served as an endogenous control. (TIF 21 kb)

Additional file 4: **Table S1.** Pepper primers used for qPCR in this study. **Table S2.** Tobacco primers used for qPCR in this study. **Table S3.** Primers used in these experiments. (DOCX 21 kb)

## Abbreviations

ChIP: Chromatin immunoprecipitation
ETI: Effector-triggered immunity
ETS: Effectors triggered susceptivity
GFP: Green fluorescent protein
hpa: H post application
LRR-RLK: Leucine-rich repeat receptor-like kinase
non-RD: Non-arginine-aspartate
ORF: Open reading frame
PAMP: Pathogen-associated molecular pattern
PTI: PAMP-triggered immunity
RSI: *Ralstonia solanacearum* inoculation
SAR: Systematic acquired resistance
TMD: Trans-membrane domain
VIGS: Virus-induced gene silencing
