# Supplementary figures and images for: CaLRR-RLK1, a novel RD receptor-like kinase from Capsicum annuum and transcriptionally activated by CaHDZ27, act as positive regulator in Ralstonia solanacearum resistance
Source: BMC Plant Biol. 2019 Jan 17;19:28. doi: 10.1186/s12870-018-1609-6 (PMC6337819; doi:10.1186/s12870-018-1609-6)

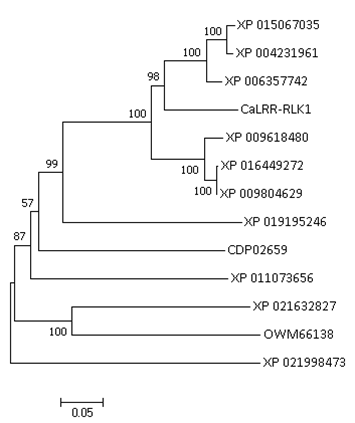

Supplement: Supplementary file 1 — Figure S1. The phylogenetic relationship of CaLRR-RLK1 protein with other plant LRR-RLKs. An unrooted neighbor-joining tree was built by MEGA 4.0. (TIF 30 kb) [file 12870_2018_1609_MOESM1_ESM.tif]

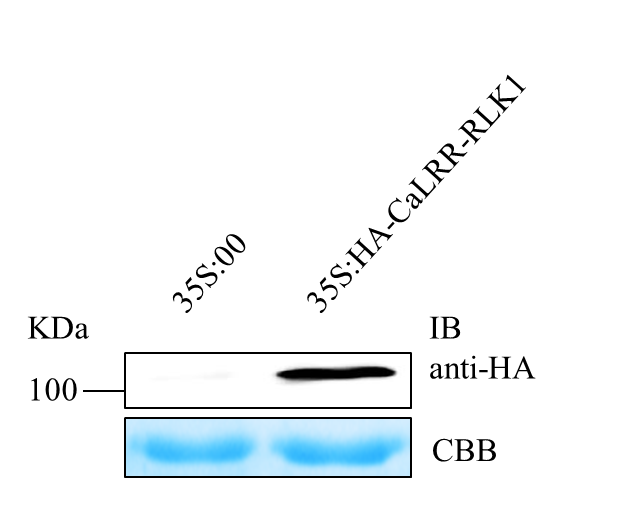

Supplement: Supplementary file 2 — Figure S2. Transient overexpression of HA-CaLRR-RLK1 in pepper leaves detected by immunoblot. (TIF 36 kb) [file 12870_2018_1609_MOESM2_ESM.tif]

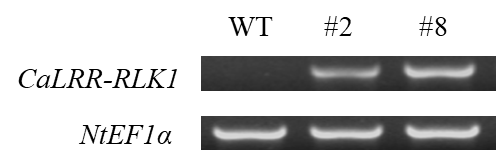

Supplement: Supplementary file 3 — Figure S3. The expression of CaLRR-RLK1 in representative T3 transgenic tobacco plants was checked by RT-PCR. Wild-type (WT) tobacco plants as the blank control, and NtEF1α served as an endogenous control. (TIF 21 kb) [file 12870_2018_1609_MOESM3_ESM.tif]
