# Supplementary material for: CaLRR-RLK1, a novel RD receptor-like kinase from Capsicum annuum and transcriptionally activated by CaHDZ27, act as positive regulator in Ralstonia solanacearum resistance
Source: BMC Plant Biol. 2019 Jan 17;19:28. doi: 10.1186/s12870-018-1609-6 (PMC6337819; doi:10.1186/s12870-018-1609-6)
Supplement: Supplementary file 4 — Table S1. Pepper primers used for qPCR in this study. Table S2. Tobacco primers used for qPCR in this study. Table S3. Primers used in these experiments. (DOCX 21 kb) [file 12870_2018_1609_MOESM4_ESM.docx]

**Table S1** Pepper primers used for qRT-PCR in this study.

| **Gene** | **Accession no.** | **Forward primers (5’ to 3’)** | **Reveres primers (5’ to 3’)** |
| --- | --- | --- | --- |
| *CaLRR-RLK1* | CA00g42040 | CGGATCTTAAACCTCTGC | GCTAACACGTCTCGCTCT |
| *CaHIR1* | AY529867 | GACAAAGCTAATGAAGCATTCTAC | GGTGTCGAAGTACTGGGTTACC |
| *CaACO1* | *AB434925.1* | CCATTGTGGTCAACCTTGGC | GCATCGCTTCCTGGATTGTAA |
| *CaPR4* | AF244122.1 | CAACCCGCAGAACATCAACTGG | CCTCAAGCATCTACCGCAAGCA |
| *CaPO2* | DQ489711 | TGATTGCTTTGTTCAGGGTT | ATGATGGACCTCCAACGAGA |
| *CaNPR1* | X61679.1 | ACTTCTTCGCCGACGCCAAG | GCCAACACATTCACCAGAGCATC |
| *CaPR1* | AF348141.1 | GCCGTGAAGATGTGGGTCAATGA | TGAGTTACGCCAGACTACCTGAGTA |
| *CaActin* | GQ339766 | AGGGATGGGTCAAAAGGATGC | GAGACAACACCGCCTGAATAGC |
| *18s rRNA* | EF564281 | CCGGTCCGCCTATGGTGTGCACCGGTCGTC | GCAGTTGTTCGTCTTTCATAAATCCAAGAA |

**Table S2** Tobacco primers used for qRT-PCR in this study.

| **Gene** | **Accession no.** | **Forward primers (5’ to 3’)** | **Reveres primers (5’ to 3’)** |
| --- | --- | --- | --- |
| *NtPR2* | M60460 | TGATGCCCTTTTGGATTCTATG | AGTTCCTGCCCCGCTTT |
| *NtPR3* | X51425 | CAGGAGGGTATTGCTTTGTTAGG | CGTGGGAAGATGGCTTGTTGTC |
| *NtHSR201* | X95343 | CAGCAGTCCTTTGGCGTTGTC | GCTCAGTTTAGCCGCAGTTGTG |
| *NtHSR515* | X95342 | TTGGGCAGAATAGATGGGTA | TTTGGTGAAAGTCTTGGCTC |
| *NtEF1α* | D63396 | TGCTGCTGTAACAAGATGGATGC | GAGATGGGGACAAAGGGGATT |
| *NtActin* | U60489 | TCACAGAAGCTCCTCCTAATCCA | GAGGGAAAGAACAGCCTGAATG |

**Table S3** Primers used in these experiments.

|  | **Forward primers (5’ to 3’)** | **Reveres primers (5’ to 3’)** |
| --- | --- | --- |
| ChIP-qPCR | ACTTCCTCTGTTCGCTTTT | ATACTTCCTCCGTTTGCTT |
| VIGS | TAATAGGCCCTCCATGAGAAGAGT | CATCAAGCATACCAAAACAAACACC |
| Gene cloning | ATGGATAACATGAAAATCCAA | TTATTTGGGACGGGGAAAG |
| Promoter cloning | GATAAATACTAAAGAGGGCAAA | TGGATGGTGGGAAGATGAGA |
